# Transmissible Endosomal Intoxication: A Balance between Exosomes and Lysosomes at the Basis of Intercellular Amyloid Propagation

**DOI:** 10.3390/biomedicines8080272

**Published:** 2020-08-04

**Authors:** Anaïs Bécot, Charlotte Volgers, Guillaume van Niel

**Affiliations:** Institute of Psychiatry and Neuroscience of Paris (IPNP), INSERM U1266, Université de Paris, “Endosomal dynamic in neuropathies“, F-75014 Paris, France; anais.becot@inserm.fr (A.B.); charlotte.volgers@inserm.fr (C.V.)

**Keywords:** amyloid propagation, amyloidogenesis, exosomes, extracellular vesicles, Aβ peptide, C99, Alzheimer’s disease, lysosomes, autophagy, endosomal sorting, proteostasis network, transmissible endosomal intoxication

## Abstract

In Alzheimer′s disease (AD), endolysosomal dysfunctions are amongst the earliest cellular features to appear. Each organelle of the endolysosomal system, from the multivesicular body (MVB) to the lysosome, contributes to the homeostasis of amyloid precursor protein (APP) cleavage products including β-amyloid (Aβ) peptides. Hence, this review will attempt to disentangle how changes in the endolysosomal system cumulate to the generation of toxic amyloid species and hamper their degradation. We highlight that the formation of MVBs and the generation of amyloid species are closely linked and describe how the molecular machineries acting at MVBs determine the generation and sorting of APP cleavage products towards their degradation or release in association with exosomes. In particular, we will focus on AD-related distortions of the endolysomal system that divert it from its degradative function to favour the release of exosomes and associated amyloid species. We propose here that such an imbalance transposed at the brain scale poses a novel concept of transmissible endosomal intoxication (TEI). This TEI would initiate a self-perpetuating transmission of endosomal dysfunction between cells that would support the propagation of amyloid species in neurodegenerative diseases.

## 1. Introduction

In the process of amyloidogenesis, amyloids can arise from many different proteins and aggregate generally into long fibrils [1]. Although amyloidogenesis is required for different physiological functions [2], an abnormal accumulation and spreading of amyloids is tightly linked to the aetiology of multiple neurodegenerative diseases, including Alzheimer’s disease (AD), Parkinson’s disease (PD), and amyotrophic lateral sclerosis (ALS) [3,4]. These common features imply that similar intracellular mechanisms are involved in these pathologies and highlight the need for an improved comprehension of the cell biological processes that support accumulation and spreading of amyloids. In this review, we will mainly focus on AD and the intracellular trafficking processes of the late endosomal system and how they determine the generation, degradation, and spreading of amyloid precursor protein (APP) cleavage products that include β-amyloid (Aβ) peptides.

Alzheimer’s disease is characterized by the accumulation of proteinous soluble and insoluble amyloid aggregates and fibrils of Aβ peptides, which result from the cleavage of APP through the amyloidogenic pathway [5]. While Aβ fibrils form the characteristic extracellular amyloid plaques, the Aβ cytotoxicity is rather associated with oligomers that cause synapse loss and cell death [6]. The onset of AD manifests by a defective functioning of the endolysosomal pathway and the dissemination of amyloids throughout the brain, which is linked to the overproduction and/or defective clearance of these amyloids. While overproduction is mainly associated with familial AD (FAD), a defective degradative pathway is shared by FAD and sporadic late onset AD (LOAD), the latter representing the large majority of the cases [6].

By combining the current understanding of the endolysosomal system that is based on observations made in disease models as well as clinical observations, this review focuses on multivesicular bodies (MVBs) that form a cumulative repository of amyloids. We will review the role of MVBs at the crossroad of endosomal-autophagic-lysosomal system and how endolysosomal aberrations will affect both the biogenesis and clearance of APP cleavage products (mainly C99 and Aβ) that are newly synthesized or have re-entered the system on endocytosis. The accumulation of amyloid products can result in endosomal congestion and toxicity leading to the activation of alternative degradative pathways such as autophagy, to the secretion of the endosomal content or eventually to cell death. This release of endosomal content on the fusion of MVBs with the plasma membrane (PM) results in the secretion of exosomes and associated cytotoxic amyloid species in the intercellular environment and implies that endosomal secretion contributes to the propagation of APP cleavage products. This way, endolysosomal dysfunctions not only concern the respective cell but also neighbouring cells. We propose here that cycles of endocytosis, congestion, and secretion spread from cell to cell and introduce a novel concept of transmissible endosomal intoxication (TEI) that may be the basis of amyloid propagation in AD as well as in other amyloid-associated diseases. The aim of this review is to validate this concept and its implications by describing how alterations in MVB processing can lead to alterations in amyloid formation, lysosomal amyloid degradation, exosome release, and amyloid dissemination.

## 2. From Early Endosome to MVB: A Sorting Station for APP and Its Secretases

**Figure 1:**
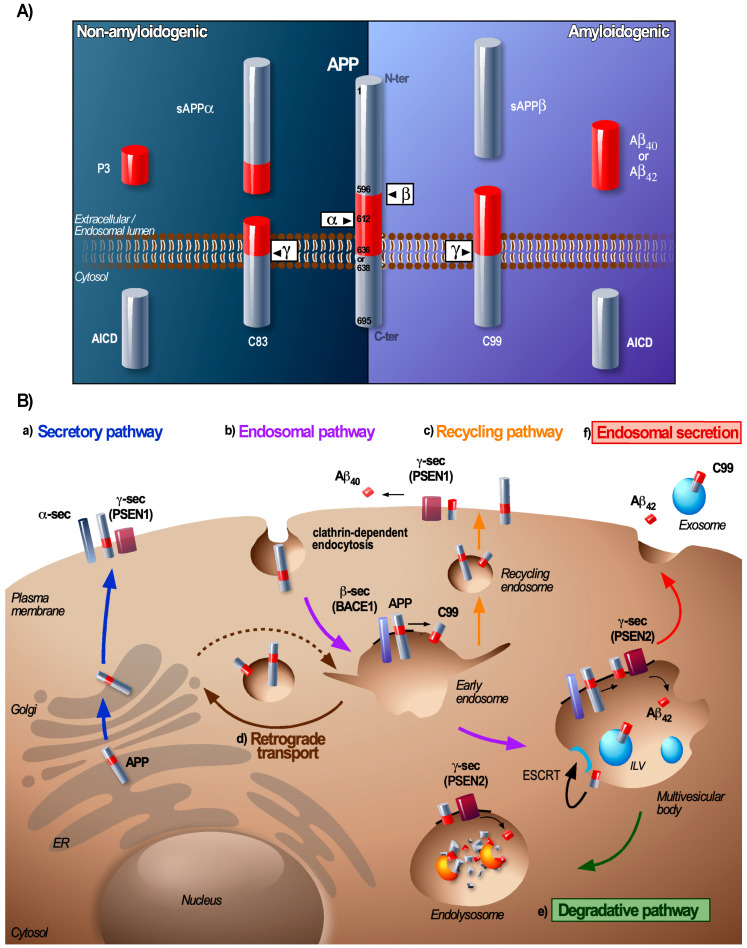
Processing and trafficking of amyloid precursor protein (APP) through the endolysosomal pathway. (**A**) Canonical processing of APP. Under physiological conditions, APP is preferentially processed through the non-amyloidogenic pathway (left panel). During non-amyloidogenic processing, APP undergoes a first cleavage by the α-secretase (mainly ADAM10) within the Aβ domain (red portion) that releases a soluble sAPPα fragment and the membrane-anchored C83 fragment (also called CTFα). The γ-secretase complex then cleaves C83 resulting in the release of a small P3 peptide and the intracellular APP intracellular domain (AICD) fragment. The amyloidogenic pathway (right panel) is responsible for the production of Aβ. Under physiological conditions, this pathway is active but only a minority of the total APP is processed this way, during AD however, the metabolism of amyloids by this pathway is increased. The amyloidogenic pathway involves a first cleavage of APP by the β-secretase (BACE1) that produces a soluble sAPPβ fragment and a membrane-anchored C99 fragment (or CTFβ). The γ-secretase complex then cleaves C99 and generates the intracellular AICD fragment and the amyloidogenic Aβ fragment, of which Aβ_40_ and Aβ_42_ are the most important Aβ species associated with Alzheimer’s disease (AD). The distinct cleavage sites on the APP sequence are indicated in black. The two canonical pathways for APP processing not only rely on different secretases but also occur at distinct cellular locations. (**B**) Trafficking of APP and its cleavage products. After synthesis, APP is translocated in the ER where its N-terminal signal peptides are cleaved. APP is then transported to the Golgi apparatus to undergo different post-translational modifications that lead to the mature form of APP. From the Golgi apparatus, APP can reach the plasma membrane via the secretory pathway (**a**) where it is either processed by the non-amyloidogenic pathway (not described here) or internalized. Upon clathrin-dependent endocytosis from the plasma membrane or via anterograde transport (dashed arrow) APP is targeted towards endosomal compartments (**b**) where processing by the amyloidogenic pathway mainly takes place. From the early endosomes, APP and C99 can be recycled to the plasma membrane via recycling endosomes (**c**) or processed to the Golgi apparatus via retromer-mediated transport (**d**) or remain in early endosomes that mature into multivesicular bodies (MVBs). The cleavage of C99 at the PM (PSEN1) or in endosomal compartments (PSEN2 mainly) generates different Aβ species, rather Aβ_40_ at the plasma membrane and Aβ_42_ intracellularly. Within MVBs, APP/C99 can be further processed or sorted onto ILVs in order to be degraded by lysosomal enzymes within the endolysosome (**e**). Alternatively, MVBs can fuse with the plasma membrane to release their luminal content; Aβ and C99/APP-associated exosomes (**f**). The localization of the APP secretases in this scheme represents the place where they are mainly catalytically active and facilitate the processing of APP.

The processing of APP and the secretases by the endosomal pathway has been extensively studied (for review, see [5,7,8]). Under physiological conditions only a minor portion of the APP is processed by this amyloidogenic pathway whereas for familial AD (FAD), this is dramatically increased [5]. Amyloidogenic processing of APP in neurons starts with a first cleavage by the membrane-associated β-secretase BACE1 (beta-site amyloid precursor protein cleaving enzyme 1), that liberates a soluble N-terminal ectodomain sAPPβ and a membrane-embedded C-terminal fragment of 99 amino acids, C99 or CTFβ [9]. This first step forms the rate-limiting step in the Aβ production. C99 can then be processed by the γ-secretase complex that is composed of four subunits (presenilin 1 or 2 (PSEN1 or PSEN2), nicastrin, presenilin enhancer 2 (PEN-2) and anterior pharynx-defective 1 (APH-1)) and the intricate intramembrane cleavage of C99 generates Aβ and the APP intracellular domain (AICD) fragment (Figure 1A) [5]. C99 and subsequent Aβ can also be potentially generated from the intermediate APP product CTFη (produced after cleavage of APP by the recently described η-secretase [10,11]). Genome wide association (GWAS)-based analyses have identified several genetic factors for LOAD (e.g., APOE4, BIN-1, PICALM, CD2AP or PLD3) to be involved in endosomal APP trafficking [12,13]. A large body of evidence demonstrates that the trafficking of APP and its proteases forms a major regulatory process of APP cleavage and that endosomes are a major site of the amyloidogenic APP cleavage [14] (Figure 1B).

Transmembrane APP and extracellular soluble Aβ enter the early endosomes (i.e., Rab5 GTPase positive compartments) when they are internalised by clathrin-mediated endocytosis [14]. For APP this process is dependent on its cytoplasmic YENPTY motif that binds adaptor proteins (AP) [15] while the endocytosis of Aβ occurs after its binding to the plasma membrane receptors [16]. BACE1, which is a type I transmembrane protein, is also endocytosed and targeted to early endosomes, but mainly by clathrin-independent Arf6-dependent endocytosis [17]. Early endosomes provide an optimal environment for aspartyl protease activity of BACE1 and thus for the processing of the full-length APP into C99 [9].

Generally, amyloid-associated diseases display a similar phenotype characterized by an increased number of enlarged early endosomal compartments, an aberrant formation and processing of MVBs, an accumulation of autophagosomes, and lysosomal dysfunction [18,19,20]. Brain biopsies of AD patients have revealed that these anomalies can appear as much as decades before the cognitive symptoms of the disease become manifest [21,22,23,24]. From a cell biological perspective, it has been suggested that the enlargement of early endosomes is caused by the accumulation of C99 within endosomes that leads to the pathological activation of the GTPase Rab5 through interaction with the Rab5 effector APPL1 (adaptor protein phosphotyrosine interacting with PH domain and leucine zipper 1) [25,26]. In return, prolonged activation of Rab5 would favour APP cleavage into C99, which implies a pathological loop resulting in lysosomal acidification defects, synaptic and cognitive alterations, and cholinergic neurodegeneration [27]. Another study suggests that the effect of C99 on early endosomes is caused by a dysregulation of the lipid homeostasis and relies on its ability to bind the lipid flippase transmembrane protein 30A (TMEM30A) that translocates phospholipids from the outer to the inner leaflet of the lipid bilayer [28]. Yet overall, it is clear that the enlargement of early endosomes and the accumulation of C99 lead to endosomal dysfunctions that will affect APP cleavage product transport and endosomal maturation. Of note, these early endosomal alterations seem to be particularly associated with familial AD where they emerge as a consequence of specific mutations in *APP* and potentially *presenilin 1* and *2* [29,30]. In sporadic AD (SAD, also known as LOAD) associated with aging, genetic, and environmental risk factors, the mechanisms underlying the endosomal defects are less well understood, although several susceptibility risk factors are implicated in the endosomal pathway. It has, however, been shown that in the brains of patients with SAD, the levels of C99 are increased [26,31,32], which indicates that it could be linked to endosomal dysfunction. Therefore, factors that stimulate the endosomal targeting of APP and BACE1 would hereby be able to enhance the cleavage of APP. For instance, increased cholesterol levels are associated with AD and cholesterol enriched microdomains at the plasma membrane have been shown to cluster APP and BACE1 in membrane microdomains, associated with a rapid co-endocytosis of APP and BACE1 to early endosomes, promoting APP cleavage [33,34].

Early endosomes act as a central sorting platform where cargoes can be retrieved for recycling to the PM or to the Trans-Golgi Network (TGN) whereas other cargoes remain sequestered within early endosomes that mature into MVBs [35] (Figure 1B). Aberrant functioning of proteins that regulate recycling or retrograde transport has been identified as a risk factor for AD [12]. In this regard, one major player implicated in the endosome-to-TGN recycling of APP and C99 is the sortilin-related receptor SORLA [36]. SORLA is highly expressed in the brain and several genetic variants are associated with a higher risk of developing SAD [12,13]. Furthermore, a decreased expression of genes that encode retromer components (e.g., vacuolar protein sorting Vps 35 and Vps26 responsible for retrograde transport of APP, C99, and BACE1) and Rab11A (involved in endosomal recycling), was also observed in AD patients [37]. Early endosomes then form a central hub where APP and BACE1 can coincide to produce C99 and aberrations of substrate and enzyme retrieval from this compartment can lead to an increased C99 production [38,39].

The γ-secretase complex, which processes the C99 into Aβ is assembled in the secretory pathway and is active at the PM and in endolysosomal compartments [40,41,42,43]. There is a distinct localisation for γ-secretase complexes containing PSEN1 or PSEN2 that are generally localized at the PM and early endosomes (PSEN1) and within late endolysosomal compartments (PSEN2) [43]. Consequently, there are distinct Aβ-peptides produced at the PM or in endosome/lysosomes (Figure 1B). Changes in γ-secretase transport with in particular its retrograde transport also have been shown to contribute to the generation of Aβ_42_ [43,44]. Interestingly, overexpression of the retromer componnt Vps35 in an AD mouse-model completely rescued the phenotype (as it normalized the Aβ formation, synaptic and cognitive functions as well as neuro-inflammation) [45]. To the contrary, deletion of *SORLA* in mice significantly increases the levels of Aβ and reduced levels of neuronal SORLA have been associated with mild cognitive impairment [38,46]. This suggests that cells can regulate the amyloidogenic processing of APP by attuning the different trafficking pathways of APP, BACE1, and the γ-secretase and that any impairment of these pathways can enhance the amyloidogenic cleavage of APP.

## 3. At the MVB: The Coordinated Conveyance of Processing and Sorting of APP

The generation of intraluminal vesicles (ILVs) within MVBs forms a key element during the maturation of the endolysosomal system. The generation of ILVs and the sorting of specific proteins and lipids onto ILVs is under the control of various sorting machineries [47]. Detailed analyses of the MVB content by fluorescence microscopy and electron microscopy revealed that ILVs can contain APP and BACE1 [48,49,50]. The first APP cleavage product that was reported on ILVs that had been released as exosomes was Aβ [51] but the current consensus is now that only a small fraction of Aβ is released in association with ILVs and thus that most of the Aβ is released in a free form which has the potential to associate with exosomes extracellularly [52,53,54]. Multiple studies based on in vitro or in vivo AD-models have demonstrated that other amyloid cleavage products such as APP, APP C-terminal fragments C99 and C83 (APP-CTFs), and AICD can also be present on exosomes and in particular C99 seems to be highly enriched on ILVs [55,56,57,58,59].

The biogenesis of ILVs involves several interconnected mechanisms starting with membrane reorganisation and the generation of lipid-protein microdomains that facilitate cargo protein clustering into the ILV [47]. In this context, a loss of function of the CD2-associated protein (CD2AP, a risk factor for AD) that controls the clustering of cargoes at the limiting membrane of the MVB, results in a reduced APP sorting onto ILVs [60]. This inward membrane budding involves machineries belonging to the endosomal sorting complex required for transport (ESCRT)-dependent or -independent pathways. The ESCRT-dependent pathway relies on an ubiquitin-dependent recruitment of four cytosolic ESCRT subcomplexes (ESCRT-0, I, II, and III) and associated proteins (accessory ESCRT-II ALG-2 interacting protein X (Alix), vacuolar protein sorting-associated protein 1 (VTA1), and ATPase vacuolar protein sorting 4 (Vps4)) to the endosomal membrane [47]. Several studies demonstrate that the APP sorting occurs in an ESCRT-dependent manner as APP mutants lacking an ubiquitination site and depletion of Hrs (ESCRT-0) or Tsg101 (ESCRT-I) prevented APP/C99 sorting into ILVs, leading to APP accumulation in the MVB limiting membrane [49,61,62]. This process is also regulated by the lipid composition of MVBs as phosphatidylinositol-3-phosphate PI3P (a phospholipid required for Hrs recruitment) deficiency reduces APP sorting into ILVs [48].

The involvement of the ESCRT machinery does not exclude distinct mechanisms to be involved in the sorting of the same cargo protein according to the cellular context [63]. With respect to APP, an impaired generation of PI3P and a disrupted ESCRT dependent sorting result in the release of APP as well as C99 in association with exosomes that have a distinct lipid composition [49,64]. This suggests that alternative sorting mechanisms are at play. Amongst these alternative sorting machineries, the semi-ESCRT-dependent syntenin-Alix- pathway have been involved in the biogenesis of ILVs. Here, syntenin and Alix generate a bridge between the sorted cargo and ESCRT-III components via the recruitment of charged multivesicular body protein 4 CHMP4 (ESCRT-III) and the ATPase Vsp4 [65]. Other ESCRT-independent pathways for ILV biogenesis involve specific proteins and lipids, such as tetraspanins [66] and sphingolipid ceramides [67], but these pathways could act synergistically as tetraspanins sorting to ILVs would depend on semi-ESCRT-dependent pathways [65,68]. The tetraspanin TSPAN6, which is found to be increased in the prefrontal cortex of AD patients, interacts with syntenin and regulates the sorting of APP-CTF into ILVs [69,70]. The exact role of TSPAN6 in the ILV generation is however still controversial and we cannot exclude that it is part of a specific sorting mechanism of APP and/or C99 to a subpopulation of ILVs fated for secretion as exosomes. In addition to tetraspanins, there are also several lipids implicated in ESCRT-independent ILV biogenesis. Ceramides which are sphingolipids produced via hydrolysis of sphingomyelin by neutral type II sphingomyelinase (nSMase), have the potential to create lipid microdomains and to induce membrane budding within MVBs [67]. Despite significantly increased ceramide levels in the cerebrospinal fluid of AD patients [71,72] and their potential role in Aβ oligomerization [73], it has not been demonstrated that ceramides are directly involved in the APP sorting. Other lipids, such as cholesterol, can also potentially affect APP and C99 sorting to ILVs for example via the direct interaction of cholesterol with APP [74,75].

**Figure 2:**
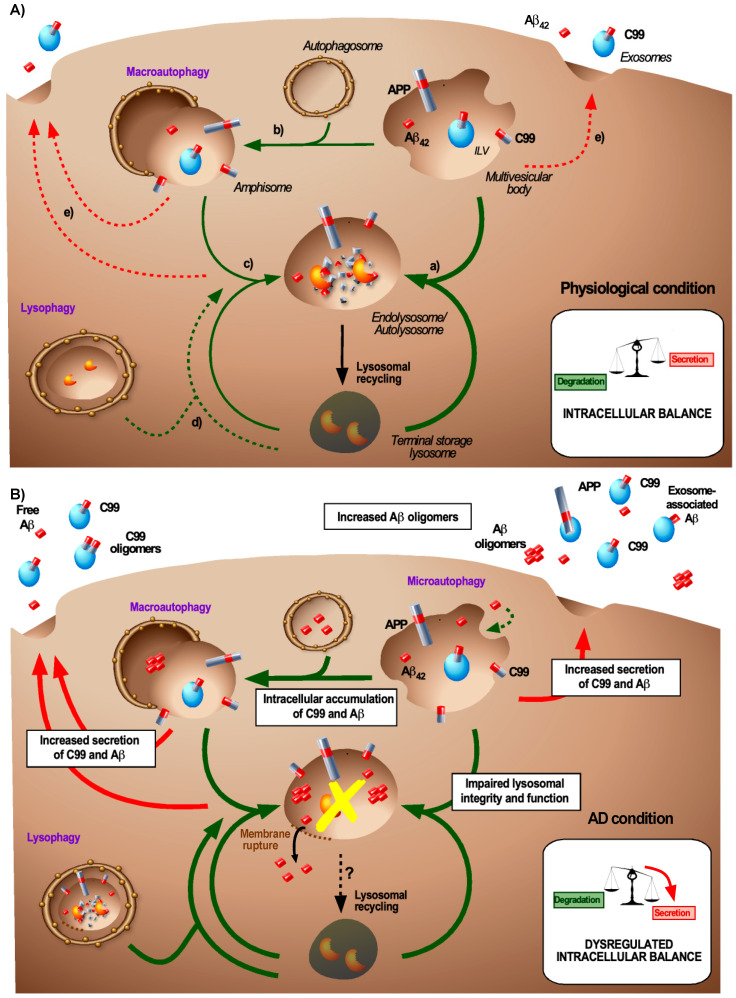
Balance between APP product degradation and secretion under normal and AD-associated conditions. (**A**) Intracellular balance for the generation and degradation of APP products under physiological conditions. Under physiological conditions, MVBs that may contain APP/C99/Aβ_42_ can have several fates, all leading to the degradation of their content by lysosomal enzymes: (**a**) MVBs fuse directly with terminal storage lysosomes to form degradative endolysosomes or (**b**) they reach the autophagic pathway by a subsequent fusion with autophagosomes that forms amphisomes and (**c**) terminal storage lysosome (TSL) to generate autolysosomes. Under physiological conditions, the selective clearance of excessive lysosomes through lysophagy (**d**) or the release of the MVB-, amphisome-, or endolysosome/autolysosome content into the extracellular space (**e**) only represents a minor clearance pathway. (**B**) Intracellular balance for the generation and degradation of APP products during AD-related conditions. During AD, several intracellular changes are thought to accumulate (as indicated in the white boxes) to a deregulation of the balance between degradation and secretion of APP/C99/Aβ_42_. The two main early changes are the accumulation of endocytosed or de novo produced C99 and Aβ_42_ in endosomal and autophagic compartments, which likely occurs concomitantly with lysosomal dysfunction. Additionally, the lysosome integrity may also be impaired upon amyloid accumulation. This can lead to membrane rupture and the release of Aβ in the cytosol and then enhances lysophagy to clear damaged lysosomes. To counteract this toxic C99/Aβ_42_ accumulation, these species can be secreted, either or not in association with exosomes.

During the generation of ILVs, cytosolic material can be non-specifically processed into the lumen of the ILV but it can also be sorted by a specific sorting mechanism called microautophagy [76]. Microautophagy, which is mainly studied in yeast vacuole, is an active pathway for the processing of cytosolic proteins to MVBs of mammalian cells [77]. This pathway relies on the recognition of KFERQ domain of protein cargo by the chaperone heat shock protein Hsc70 and involves autophagy-related proteins such as Atg12-Atg3 [78], LC3 [79], and ESCRT-related components such as Alix, tumor susceptibility gene protein Tsg101, and Vps4 [77]. So far, this process has been associated with cytosolic protein (e.g., GAPDH) sorting [77,78,79] and represents a first wave of degradation/nutrient recycling during starvation before the occurrence of bulk macroautophagy (see chapter 4 for different types of autophagy) [80]. This mechanism would be of crucial importance for the degradation of amyloids present in the cytosol. These cytosolic amyloids derive from cytosolic proteins such as Tau [81] but also from endocytosed amyloids that reach the cytosol after membrane leakage of endolysosomes (Figure 2, AD condition). APP is a transmembrane protein with a KFERQ-like motif in its cytosolic domain. Deletion of this motif increases the generation and release of APP-CTFs, which indicates that this sequence is important for the regulation of APP processing [82]. Its relevance to ILV sorting during microautophagy, however, remains to be assessed.

Together these studies demonstrate that the sorting of APP onto ILVs is directly linked to its cleavage. This holds true for a large number of other cargoes including physiological amyloids (i.e., premelanosome protein PMEL) that require secretase cleavage to be sorted onto ILVs [66,83]. This sorting to ILVs allows physical segregation of sorted proteins and lipids from the limiting membrane of MVBs. In this context we can wonder whether APP, once on ILVs, is segregated from its proteases or co-sorted with them. In the first case, sorting would prevent the processing of APP while the second case would confer to ILVs the status of a processing platform. Exosome analyses revealed that exosomes besides C99 can also contain BACE1 and apparently an incomplete γ-secretase complex [57,58]. The precise sorting mechanism of BACE1 is not known but it has been shown that ceramides can promote the stabilization of BACE1 and hereby support its activity and the generation of C99 [72]. The γ-secretase complex can be incorporated in tetraspanin enriched microdomains, favouring the amyloidogenic cleavage of C99 but how this affects its sorting remains to be investigated [84,85]. Although we cannot exclude that the cleavage of APP by BACE1 can occur on ILVs, it is likely that the BACE1 mediated cleavage of APP takes place before their sorting as C99 can accumulate in early endosomes with no or few ILVs [61]. The inhibition of APP sorting to ILVs enhances the generation of Aβ [48,61,69], suggesting that if C99 is not sorted to ILVs, it is cleaved by γ-secretase to generate Aβ. In concordance, the impairment of γ-secretase cleavage and in particular PSEN2 stimulates the sorting of APP-CTFs onto ILVs and exosomes [49,58]. This indicates that the sorting of APP-CTFs to ILVs occurs preferentially before γ-secretase cleavage and is in line with evidence that suggests that PSEN2 acts at a later stage within endolysosomes once ILVs already have been formed [43]. However, we cannot rule out the generation of different ILV subpopulations containing either APP products or the secretases, which are hereby kept separated.

In this context one can wonder whether the sorting of APP/C99 to ILVs is beneficial or detrimental. When the sorting of C99 to ILVs would impair the Aβ formation and target C99 for degradation it would be beneficial. However, AD neurons show a large endosomal accumulation of APP products (Aβ and C99) and in particular the accumulation of C99 has been found to occur at very early stages even before the appearance of any detectable Aβ plaques [86], suggesting that C99 (possibly in association with ILVs) might be involved in the onset of AD. MVBs can also accumulate extracellular amyloids such as Aβ and Tau upon endocytosis. Such an accumulation of endogenous and exogenous amyloid would impair the endolysosomal system and lysosomal degradation, favouring accumulation, aggregation, and associated cytotoxicity of amyloid oligomers [81,87,88,89]. This accumulation also damages the endolysosomal membrane resulting in the release of amyloids in the cytosol, which contributes to cytotoxicity and the propagation of amyloids [90,91,92]. In this context, the ESCRT machinery is key for membrane repairing processes [93] and it was recently shown that a compromised function of the ESCRT machinery promotes the release and propagation of Tau protein [94].

All in all, MVBs play a dual role in the homeostasis of APP cleavage products. They provide an optimal environment for a well-coordinated amyloid processing. In particular, sorting to ILVs may offer an additional level of control for APP processing by physically separating APP cleavage products and proteases between the limiting membrane of the MVB and ILVs. However, MVBs also accumulate amyloids generated in situ and amyloid species upon endocytosis that would impair their lysosomal degradation.

## 4. Relevance of Autophagy in Amyloid-Associated Endosomal Dynamics

Autophagy is a major regulatory process that controls the cellular homeostasis and is based on the recycling of cytosolic cargo proteins and organelles upon lysosomal degradation. In addition to starvation, other stressful conditions that cause organelle dysfunction such as the accumulation of misfolded proteins upregulate autophagy [95]. Classically, three autophagic pathways have been identified: microautophagy, chaperone-mediated autophagy (CMA), and macroautophagy [95,96]. Macroautophagy is the most prevalent and most important pathway for the degradation of aggregated proteins (therefore also known as aggrephagy) [97]. Macroautophagy is a multi-step process in which aggregated proteins or defective organelles are captured by double-membrane organelles called autophagosomes. In turn, these autophagosomes then fuse with lysosomes hereby generating autolysosomes [98] (Figure 2). The complex molecular machinery that drives macroautophagy is well-described and involves several autophagy-related gene (Atg)-coding proteins [98]. A key step in the autophagosome formation is the microtubule-associated protein 1 light chain 3 (LC3) lipidation through the conjugation of LC3-I with phosphatidylethanolamine (PE) to form LC3-II [98].

There is a substantial body of evidence showing a high degree of overlap between macroautophagy and endolysosomal pathways, in particular with the ESCRT-dependent pathway for ILV biogenesis. Firstly, these pathways are both dependent on PI3P and the ubiquitination of cargo proteins. Secondly, cells depleted for the ESCRT components Tsg101 and Vps4 [99] or Alix [78] reveal an impaired formation of MVBs as well as autolysosomes. Moreover, the ESCRT subunit CHMP2A is required both for phagophore closure and for ILV budding [100]. Finally, TSPAN6 overexpression induces enlarged MVBs and impairs autophagy by inhibition of the fusion between autophagosomes and lysosomes [69]. Additionally, some well-known autophagic proteins are also found to control endosomal processes. For example, Atg5 besides being involved in microautophagy (see chapter 3 for the description of microautophagy) also appears to be important for autophagosome formation as well as ILV secretion [101]. Autophagic and endosomal pathways can also converge when maturing autophagosomes fuse with MVBs to generate amphisomes resulting in autophagosomal degradation [99] (Figure 2), yet the exact mechanism for this form of degradation is still poorly defined. It is however known that this fusion involves the GTPase Rab11 and SNARE proteins. Although Rab11 is not required for autophagic processing, it is required for MVB-autophagosome fusion and a loss of Rab11 leads to the accumulation of autophagosomes and late endosomes and impairs the maturation of autophagosomes [102].

This overlap between endocytic and autophagic pathways seems crucial for the C99- and Aβ-associated AD pathogenesis. Studies based on the induction or the inhibition of autophagy revealed that APP and the APP cleavage products (C99 and Aβ) can be degraded by autophagy [89,103,104] (Figure 2, physiological condition). The autophagic degradation of C99 has been shown to be partly dependent on Atg5 [105] and plasma Atg5 levels are increased in AD patients [106]. Moreover, it was suggested that the complex formed by adaptor protein 2 (AP2) and phoshphatidylinositol binding clathrin assembly protein (PICALM) acts as a cargo receptor for the recognition and the transport of C99 from the endocytic pathway to autophagosomes, through the binding of AP2-PICALM to LC3, leading to the degradation of C99 [107]. This mechanism is particularly interesting because PICALM also has been identified as a genetic risk factor for AD [13]. Yet PICALM may have pleiotropic roles in the APP processing notably by controlling endocytosis and lysosomal targeting of γ-secretase [108]. As a clearance mechanism, macroautophagy can be stimulated by extracellular Aβ [109] and autophagy appears as a natural pathway to clear amyloid aggregates from the cytosol. In AD, this pathway is involved in the clearance of Tau [110] and can also affect the clearance of APP, C99 and Aβ in several different ways. APP cleavage products that are present in MVBs can be targeted to the autophagic pathway through the generation of amphisomes [99]. The cytotoxic accumulation and aggregation of Aβ in endolysomes can also permeabilize the lysosomal membrane and lead to the release of Aβ in the cytosol (Figure 2, AD condition) and hence the subsequent clearance is likely to require aggrephagy [111]. Finally, lysophagy that allows the clearance of damaged lysosomes [112] would be of prime importance during AD to prevent the accumulation of damaged lysosomes (Figure 2, AD condition). Thus, hereby macroautophagy can provide with additional means for the clearance of APP products, either through their direct targeting to autophagosomes or the clearance of damaged lysosomes.

In addition to macroautophagy, both CMA and microautophagy are important processes that control the autophagy of proteins rather than organelles. CMA occurs in LAMP2a positive compartments that likely correspond to endolysosomes or autolysosomes. During CMA, proteins that carry the KFERQ motif are recognized by Hsc70 resulting in a protein unfolding and in processing by LAMP2a to the lumen of the autophagic compartment [76]. The recognition of KFERQ motif is also involved in microautophagy and MVB formation (see chapter 3 for the description of microautophagy) [76]. Of note, as microautophagy involves LC3 sorting to acidic compartments, it is somewhat difficult to distinguish this process from bulk macroautophagy [80]. Although, many neurodegenerative disease-associated pathogenic proteins, such as Tau, α-synuclein, and huntingtin, are degraded by CMA [113,114,115], it is not known if this also applies to APP cleavage products. However, the presence of the KFERQ-like motif in the C-terminus of APP makes it likely that APP or its products can act as a substrate for CMA or microautophagy. This is especially likely for the AICD, which is released into the cytosol upon γ-site APP cleavage [82].

The importance of autophagy for the prevention of disease is further substantiated by the fact that brain biopsies from early-stage AD patients show an abnormal accumulation of immature and non-degraded autophagic compartments (autophagosomes, amphisomes, and autolysosomes) around amyloid plaques [24]. Although it is not completely clear whether this accumulation results from an increase in the autophagosome formation or a decrease in their degradation there are several lines of evidence that indicate this is caused by a defect in lysosomal fusion and proteolysis. On one hand, there is evidence showing an increased expression of autophagic genes during AD and it seems that Aβ by itself can induce autophagy via the initiation of the AMP-activated protein kinase (AMPK) pathway [109,116]. On the other hand, observations based on AD-mouse models and AD patients reveal a hyperactivation of the mTOR pathway, which is an indirect suppressor of autophagy [117,118,119]. However, the actual extent of the involvement of autophagy in the aetiology of AD is still a matter of debate. A recent transcriptomic study may reconcile these divergent data as it identifies AD as a progressive autophagic pathology, characterized by opposing phenotypes that manifest over the course of the disease [116]. At an early stage of disease, pyramidal neurons show an upregulation of a large number of autophagy genes essential for autophagy initiation, autophagosome formation, fusion, and lysosomal proteolysis. Interestingly, this upregulation is correlated with an increased expression of transcription factor EB (TFEB), which is the main transcriptional regulator of autophagy [116]. At a later stage of the disease, these neurons show an inhibition of the autophagic flux, which is characterized by the accumulation of autophagic substrates, of p62 and LC3-II and by an increased size and number of autolysosomes [116]. Importantly, and in concordance with the endosomal pathology, the early accumulation of C99 in autophagic compartments would contribute to autophagic defects [89] suggesting a self-sustaining relationship between autophagic impairments and the accumulation of APP cleavage products.

To conclude, endosomal and autophagic pathways are strongly interconnected pathways that mediate both the clearance of extracellular and intracellular APP cleavage products. This tight relationship between endosomal and autophagic pathways implies that endosomal defects can cause autophagic defects and vice versa. Moreover, intrinsic and external alterations such as the amyloid accumulation in AD can affect both pathways and this is especially likely to converge at the lysosomes.

## 5. Lysosomes: Generation and Degradation of Aβ

Lysosomes terminate both the endocytic and autophagic pathway and accommodate the degradation of MVBs and the content of autophagosomes, respectively (Figure 2, physiological condition). Their degradative function is ensured by acid hydrolases, the vacuolar ATPase (v-ATPase) pump, and by highly glycosylated LAMP proteins [120,121]. This lysosomal degradation aims at avoiding the accumulation of dysfunctional or toxic cellular components but also at providing disassembles macromolecules that then serve as trophic support. Lysosomes also act as a signalling hub and hereby regulate cell growth and autophagy in a mammalian target of rapamycin complex 1 (mTORC1) dependent manner [122]. Finally, some specialized cells (e.g., melanocytes, lymphocytes, macrophages, and epithelial cells) also contain lysosome related organelles that participate in specialized functions. One such example is the production of functional amyloid fibrils by melanocytes that serve as a scaffold for the melanin synthesis (Box 1) [123].

Box 1 **Comparison of functional and pathological amyloids.**

Amyloidogenesis is closely associated with pathological situations such as neurodegenerative diseases where amyloid accumulation is linked to synaptic loss and cell death. Yet under physiological circumstances, the very same amyloids, such as Aβ, can also be produced and can serve a plethora of physiological functions: They are implicated in synapse formation and plasticity, learning, memory formation, neuroprotection, and low levels of Aβ can protect against infections and aid synapse function [124,125]. This suggests that, at least for Aβ, amyloid toxicity rather relies on the capacity of cells to maintain the Aβ homeostasis than on its production per se. This notion assigns Aβ as a potentially physiological amyloid and places the endolysosomal system at the heart of this regulation. A relevant manner to understand how a situation becomes pathological is to learn from a physiological situation where specialized cells control amyloidogenesis in a well-coordinated manner.

In this context, the model based on pigmented skin cells provides with a consistent model to study the generation of amyloids as it involves similar endosomal compartments, trafficking processes, and enzymatic processing pathways as those involved in the biogenesis of functional amyloids. Pigmented skin cells such as melanocytes produce amyloid fibrils from the premelanosome protein (PMEL) in lysosome related organelles called melanosomes, these functional amyloid fibrils then serve as a scaffold for the melanin synthesis [123]. PMEL is a type I transmembrane protein that is subjected to a similar trafficking as APP and is processed into amyloid peptides in the endolysosomal system. In these compartments, PMEL undergoes sequential cleavage involving β-[126] and γ-secretases [66] and aggregates into amyloid fibrils in an apolipoprotein E (ApoE) isoform manner. Importantly, mutations that affect PMEL processing can lead to the generation of pathogenic species [127].

Therefore, there are several checkpoints that govern an immaculate processing. The first that is common to both amyloids is that their production takes place in enclosed intracellular compartments, which restricts their processing and prevents potential leakage and cytosolic toxicity. A second process that secures PMEL processing is based on the sorting of PMEL cleavage products that contain an amyloidogenic domain onto ILVs. Sorting defects lead to endolysosomal aberrations, which indicates that the segregation of amyloidogenic fragments from the limiting membrane of the compartment prevents membrane damage [66]. It is highly likely that the same holds true for C99 although its sorting to ILVs is based on a different sorting mechanism.

For PMEL, the sorting of the amyloidogenic fragment onto (ApoE containing) ILVs leads to the generation of amyloid fibrils from the surface of the ILVs [128]. ApoE is a key regulator of Aβ fibrillation and aggregation into plaques [129]. The release of PMEL amyloids in compartments containing regulators of its fibrillation likely represents a third checkpoint to avoid cytotoxicity. In the brain, ApoE and Aβ find their origin in different cell-types and only encounter each other in the extracellular space upon their release and thus rendering a similar partnership unlikely.

Defects in PMEL-luminal domain sorting that occur in the absence of CD63 cause it to be targeted to lysosomes along with PMEL-CTF, hence it seems that this lysosomal clearance provides additional means to maintain the amyloid homeostasis. For APP however, it is precisely this lysosomal targeting that would favour Aβ production. Hence, it is of major importance to investigate the regulatory machineries that control the targeting of APP cleavage products towards the endolysosomes. Comparison of the analogies and differences between the generation of physiological and pathological fibrils may provide essential insights and can shed a new light on the processes that underlie the generation of pathological amyloids.

The latest advances in the understanding of the endolysosomal system reveal that the term lysosome covers a heterogeneous population of compartments [130]. The term lysosome can refer to (i) late MVBs, (ii) terminal storage lysosomes (TSLs), (iii) endolysosomes that have resulted from the fusion of MVBs with TSLs or to (iv) autolysosomes generated by the fusion of autophagosomes or amphisomes with TSLs (Figure 2, physiological condition). Each of them has distinct features and functions. Terminal storage lysosomes are known as storage and non-degradative compartments that are characterized by a neutral pH and inactive hydrolases [130]. Endolysosomes (often referred to as late endosomes) and autolysosomes form degradative compartments characterized by the presence of active acid hydrolases and by an acidic pH in their lumen. Lysosomal material from these endolysosomes and autolysosomes, can be recycled in order to replenish the lysosomal pool (Figure 2, physiological condition). Autolysosome reformation allows the recycling of the lysosomal membrane from autolysosomes by tubulation and pinching [131]. This process depends on mTOR signalling, the combination of various phosphoinositides, SPG proteins, and wasp homology-associated protein with actin, golgi membranes, and microtubules (WHAMM) and kinesin to extrude from protolysosomes [132]. Lysosomal recycling from endolysosomes likely involves a similar process and requires the activity of the phosphatidylinositol-3-phosphatase-5 kinase PIKfyve complex that drives membrane tubulation [133]. These recycling pathways are of major importance to maintain the degradative activity of the cell that continuously generates MVBs and autophagosomes. Dysfunctions in the lysosomal reformation have been involved in some neurodegenerative diseases such as Parkinson’s disease [134,135], the relevance of this pathway in AD, however, remains elusive.

With regard to the processing of APP, endolysosomes and autolysosomes allow degrading the near-totality of C99 and controlling the Aβ level. The main lysosomal proteases involved in C99 and Aβ degradation belong to the family of cathepsins [89,136]. Despite their ability to degrade APP cleavage products, a recent study shows that late endosomes and lysosomes can also act as the main site for the generation of intracellular Aβ [43]. The γ-secretase complex containing PSEN2 is mainly restricted to late endosomes/lysosomes due to its direct interaction with the adaptor protein AP-1 and is responsible for the production of longer Aβ (Aβ_42_ and Aβ_43_) [43]. These data suggest that the generation of intracellular aggregation-prone Aβ_42_ requires the fusion of C99-containing MVBs with TSLs to generate Aβ in endolysosomes. However, C99 and γ secretase should be localized within the same membrane micro domain, which is not the case when C99 is associated with ILVs. Strikingly, this same study reveals that the AD-associated mutations of *PSEN1*, leads to the redistribution of PSEN1 to late endosomes/lysosomes resulting in a PSEN2-like localization [43], which strengthen the role of endolysosomes in the generation of an intracellular pool of Aβ. It is likely that this lysosomal production of intracellular Aβ would be advantageous to the cell as it results in the colocalization of amyloids with hydrolases that are able to rapidly degrade them. These subtile checkpoints in the generation of these potentially toxic amyloids are reminiscent to the mechanisms used by pigment cells to generate functional amyloids in a safe manner (Box 1) [123].

As mentioned above, AD-related endolysosomal and autophagic aberrations are, i.e., defined by a lysosomal dysfunction and by the intracellular accumulation of APP cleavage products. The integration of several lines of evidence supports a model that is based on two successive stages of lysosomal dysfunction in FAD and SAD. The first characterized by an increased lysosomal biogenesis resulting in an elevated number of lysosomes in the brain and/or an increase of lysosomal proteins (Cathepsin D, LAMP-1) in the cerebrospinal fluid. This stage likely represents an act of self-defence to eliminate accumulating toxic aggregates and/or to compensate for long-term lysosomal decay that is associated with brain aging [7]. The second stage is characterized by an impaired lysosomal degradation that involves the accumulation and enlargement of lysosomal compartments (i.e., endolysosomes and autolysosomes), a reduction of the lysosomal proteolytic activity, and the lysosomal accumulation of C99 and Aβ. This stage is clearly associated with the disease and studies based on FAD and SAD brain tissues identified the impaired lysosomal acidification as one of the most profound hallmarks of the pathology [7]. PSEN1 can control lysosomal acidification by transporting v-ATPase V0a1 subunit to the lysosomal membrane and its deletion leads to an elevated lysosomal pH and an impaired transport of amphisomes and endolysosomes [137,138]. Hence, it is likely that this mechanism is implicated in FAD cases where PSEN1 is mutated. v-ATPase deficiency can also cause an impaired Ca^2+^ homeostasis, which is also linked to a defective lysosomal function [139]. The lysosomal generation of Aβ_42_ can also lead to lysosomal dysfunction by the creation of pores in the lysosomal membrane resulting in lysosomal leakage and the cytosolic accumulation of Aβ [140]. Moreover, the accumulation of Aβ and C99 in endolysosomes can also trigger other endolysosomal alterations such as the inhibition of the ESCRT machinery [87] which leads to an impaired micro-autophagy and lysophagy [141] and it can induce lysosomal enlargements and a loss of hydrolase activity [30,89,142]. In sporadic cases, the exact nature of these lysosomal alterations is less clear, but an increased lysosomal permeability is associated with the apolipoprotein E *APOE4* variant and to the accumulation of C99 [143,144]. Such dysfunctions occur in particular at low Aβ levels and are exacerbated after γ-secretase inhibition which increases C99 levels, this suggests that the accumulation of C99 rather than Aβ would contribute to lysosomal dysfunction [30,89].

Overall, at an early stage of AD, the concomitant accumulation of amyloid products and endolysosomal and autophagic defects will certainly fuel each other but it is still unclear which one initiates this self-perpetuating process. Neurons are extremely sensitive to endolysosomal and autophagic pathway aberrations due to their incapacity to dilute toxic materials by division. These aberrations, conceivably combined with the intrinsic vulnerability of neurons to accumulate aggregates are likely to underlie the pathophysiology of AD. One way to cope with this limited degradative capacity would be to redistribute materials that are destined for degradation over neighbouring cells, a process covered by the concept of the proteostasis network [145] and involves intercellular distribution of exosomes [146].

## 6. Exosomes: Clearance and Dissemination of Amyloids

Exosomes belong to the family of extracellular vesicles (EVs) that are small nanosized vesicles characterized by a lipid bilayer. EVs refer to vesicles that derive from budding from the plasma membrane (microvesicles; 50 nm-1 µM) or ILVs that are secreted by the fusion of MVBs with the plasma membrane (exosomes; 50–150 nm) [47]. Exosomes are constitutively produced by almost all cell types, including neurons, microglia, and astrocytes. They are enriched during their biogenesis in MVBs for specific proteins, lipids, and RNAs in a process that is dependent on the cell type and the pathophysiological conditions (see chapter 3 for ILV generation). The composition of ILVs not only relies on the sorting mechanism acting at MVBs but also on potential association with material in the extracellular environment once secreted. With regard to APP, exosomes are mainly found to carry Aβ and C99, which has led to several hypotheses about their implications in the AD pathogenesis. The numerous studies on this subject however do not allow the composition of an integrated theory. Exosomes were first described as a clearance mechanism of toxic amyloids [147,148] but are now considered as intercellular shuttles that act in autocrine, paracrine, and endocrine manners.

At early stages of AD, exosomes secretion would promote the clearance of toxic proteins that accumulate intracellularly in neurons (APP cleavage products as well as hyperphosphorylated Tau protein). As mentioned before, only a small portion of the total Aβ seems associated to exosomes [51] and this association may occur post-secretion [149]. Yet, the fusion of MVBs with the plasma membrane would not only result in the secretion of exosomes but also of soluble endosomal Aβ that would then likely represent the most important APP cleavage product that is released via the endosomal pathway. In addition to Aβ, exosomes are also found to carry C99, at present no physiological role has been attributed to C99 and under physiological conditions the vast majority of C99 is targeted for lysosomal degradation. However, when this degradative pathway is compromised and/or when C99 accumulates in neurons as is the case in AD, it can be released in association with exosomes and could become a substrate of the transcellular proteostasis network (TPN).

Once secreted Aβ is either taken up by microglia for degradation or it crosses the blood-brain barrier to reach the periphery (especially the kidney and liver) where Aβ-degrading enzymes (neprilysin (NEP), insulin-degrading enzyme (IDE), endothelial-degrading enzyme (ECE), and metalloproteases) are highly expressed [150]. Exosome secretion would also play a role in the neutralization of secreted Aβ, which is mediated by exosomal lipids such as glycosphingolipids and/or the prion protein present on exosomal surface and promotes its uptake and clearance by microglia cells [53,54,151,152]. This uptake can also introduce a conformational change of Aβ (favoured by the prion protein (PrPc) notably) into amyloid fibrils, which also serves to neutralize the toxicity of Aβ oligomers [52,152]. Indeed, the injection of exosomes from naïve cells or with a particular lipid signature prevents several Aβ-induced toxic phenotypes in AD mouse models (LTP, cognitive function, and inflammation) [53,54]. Moreover, the degradation of Aβ can also be further enhanced by exosomes that contain the proteases IDE and NEP that are released by neuronal as well as microglia cell lines [153,154,155]. The transfer of amyloids by exosomes would strengthen their role as mediators in the transcellular proteostasis network [146]. This network would allow cells to share their degradative machinery and the cargoes that are to be degraded. Hereby, the exosome transfer would be able to stimulate the autophagic flux [156,157] and support proteolysis (via the transmission of chaperone proteins) in neighbouring compromised cells [158]. Thus, exosomes would be able to contribute to the distribution of the extracellular Aβ pool over various cell populations within and outside the central nervous system (CNS), which would aid its degradation.

With aging and during the development of AD, the degradative capacity of recipient cells (neurons and microglia) tends to decrease progressively, this can lead to the intracellular accumulation of APP cleavage products and the release of exosomes that contain these products. This also implicates exosomes in the AD pathology where they function as a vector to disseminate toxic APP cleavage products [56,159,160,161]. Several lines of evidence support this detrimental role of exosomes. Firstly, the transfer of exosomes from cell-based AD models or derived from AD patients to naïve neurons/mice facilitates Tau and Aβ oligomer spreading [161,162] as well as neurotoxicity and neurogenesis alterations [163]. The compromised clearance by microglia that is induced by the transfer of exosomes from APP Swedish mutation expressing cells is also associated with the release of pro-inflammatory cytokines that could contribute to a general inflammatory state associated with AD [160]. In line, several studies have demonstrated that inhibition of the nSMase enzyme, which acts among other processes on the exosome generation, decreases the spreading of Tau and Aβ pathologies as well as other AD-related phenotypes including glial inflammation and cognitive defects [73,164]. In addition to neurons, the role of microglia in the AD pathogenesis that has been highlighted as microglial depletion was found to prevent the Tau pathology [164]. However, there is limited evidence on the relationship between exosomes and microglia in AD. So far, the effect of EVs from microglia on the metabolism of Aβ has been mainly attributed to microvesicles that would convert extracellular insoluble aggregates into more toxic soluble Aβ species [165]. Yet, the immunological activity of microglia and the contribution of exosomes to neuron-glia communication would also be able to indirectly act on the Aβ metabolism [166] by influencing AD-associated neuroinflammation [167]. In particular, exosomes could participate to the neuroinflammation associated to AD by carrying higher levels of pro-inflammatory cytokines (TNFα, IL6, IL1β) that potentially activate microglia and astrocyte [160].

## 7. The Endosomal Balance between Degradation and Secretion

Changes in endolysosomal and autophagic processing can cause a shift in cargo processing. The cell biology behind the interconnected organization of the endolysosomal and autophagic system suggests that pathways for degradation and secretion are linked. Two main processes seem to be involved in this balance, intrinsic mechanisms that constitutively target endolysosomal compartments for secretion or degradation and pathways that compensate for any impairments of the lysosomal degradative function. The latter case is particularly relevant in AD where an endosomal pool of amyloids would be secreted to alleviate the intracellular proteostasis load of the cell. MVBs are susceptible to both processes as they can be constitutively targeted for degradation or secretion and their secretion is stimulated upon endosomal congestion [168].

Among the intrinsic mechanisms, the sorting machineries involved in the ILV generation have been directly linked to the fate of MVB. The recruitment of sorting machinery at MVB is directed by post-translational modifications of the cargo to be sorted. Cargo ubiquitination, associated with ESCRT-dependent ILV formation, may favour its sorting towards lysosomes while non-ubiquitinated cargos that can be processed by ESCRT-independent and semi-ESCRT-dependent syntenin-Alix pathways are generally targeted towards the plasma membrane (e.g., MHCII and SIMPLE) [63,169]. Alterations of ESCRT-dependent sorting of APP to ILVs will lead to a massive secretion of APP-CTFs [48] suggesting that alternative sorting mechanisms are at play. The mechanism for ESCRT-independent sorting of APP products remains to be established but it may involve the syntenin-Alix pathway [69]. Interestingly, while Aβ oligomers have a higher affinity for CD63 positive exosomes [149], C99 seems to be enriched on CD63 negative exosomes [55] suggesting that distinct sorting mechanisms and subpopulations of exosomes may co-exist. Despite this predilection, both ESCRT-dependent [170] and -independent pathways [65,67] are capable of targeting ILVs to the PM leading to the release of exosomes. Other mechanisms may also promote the release of APP cleavage products in association with exosomes at the expense of their degradation, these include mechanisms under the control of TSPAN6 [69] or post-translational modifications such as phosphorylation (e.g., of annexin-2), glycosylation, and ISGylation [171,172,173].

The fate of MVBs (secretion vs. degradation) is likely linked to its spatial subcellular localization. In polarized and compartmentalized cells such as neurons, this localization is guarded by a regulated intracellular transport orchestrated by motor proteins along the cytoskeleton. In polarized neurons, MVBs and autophagosomes are found in the periphery (axons and dendrites) and require a controlled retrograde transport to reach the lysosomes that are localized in the soma [174,175,176,177]. Vesicular transport is under the control of the motor proteins dynein (retrograde transport) and kinesin (anterograde transport), which recruitment is regulated by Rab GTPases and by the formation of membrane contact sites (MCS) with other organelles including the endoplasmatic reticulum [178]. While regulators of vesicular transport have been shown to modulate APP and secretase trafficking [17] and can drive the release of exosomes [47], their implications for the transport of APP-containing endosomal and autophagic compartments remains to be established.

The balance between degradation and secretion, in particular in neurons, is highly dependent on extrinsic factors that affect the lysosome status. The pharmacological inhibition or a blockade of the lysosomal function can promote the release of exosomes and/or pathological exosome-associated proteins. For example, the alkalinisation of lysosomes, induced by chloroquine treatment or by blockade of the v-ATPase pump using bafilomycin A1, can lead to an increased release of exosomes (positive for CD63, Tsg101, and Alix) and of pathological proteins including those related to AD, such as C99, AICD, and Aβ [59,61,173,179,180,181,182]. Similarly, knockdown of the lysosomal enzyme cathepsin D leads to an increased exosome release and Aβ_42_ content [179]. Interestingly, the γ-secretase inhibitor treatment was shown to provoke lysosomal-autophagic aberrations and is associated to an increased secretion of APP-CTF positive exosomes [56,59,89].

The close connection between endolysosomal and macroautophagy also suggests that these pathways are coordinated to achieve an intrinsic balance between degradation and secretion. Several lines of evidence reinforce this and show that especially autophagic proteins are important regulators for the release of exosomes. For instance, the Atg12-Atg3 complex can interact with Alix to regulate autophagy, autophagosome-MVB fusion, and release of ILVs as exosomes [78]. It has also been demonstrated that the exophagy of pathological proteins (Tau/Aβ/α-synuclein) is increased by impairing autophagosome-lysosome fusion. An upregulation of the autophagic protein Atg5 also causes both a decreased MVB acidification and increases the release of exosomes [101]. Additionally the prion protein by interacting with calveolin-1 suppresses autophagosome formation leading to an increased exosome secretion [183]. Finally, the induction of autophagy in conjunction with the inhibition of the degradative autophagic flux stimulates secretion of Aβ, which may eventually contribute to an extracellular Aβ deposition [103]. Importantly, endosolysosomal and autophagic dysfunctions have been associated with changes in lipid composition of these compartments, which can also affect the biogenesis and release of ILVs. For example, genetic or pharmacological inhibition of the kinase PIKfyve affects the lysosomal degradation of MVBs and autophagosomes and increases the release of an exosome subpopulation whose content is enriched for autophagic proteins LC3 and p62 [184]. Similarly, the inhibition of Vps34, the enzyme responsible for PI3P formation, leads to the secretion of exosomes abnormally enriched for APP-CTF as well as p62. Interestingly, these exosomes appear to be particularly enriched for BMP, a lipid that is abundant in endolysosomes and under physiological conditions almost absent in exosomes [64]. At a cellular basis, AD and other pathologies such as the Niemann Pick disease type C is characterized by lysosomal dysfunction and by alterations in both exosome release and composition. Several actors that regulate the balance between degradation and secretion are altered in AD patients, examples hereof are the increased plasma Atg5 levels and the selective deficiency of PI3P in several brain regions (prefrontal and entorhinal cortex) [48,106].

In conclusion, AD seems to be associated with a dysregulation of the balance between lysosomal degradation and exosome secretion (Figure 2, AD condition). This balance would lead to the secretion of APP cleavage products that are either or not associated with exosomes when the endolysosomal system is impaired due to the accumulation of APP cleavage products.

## 8. Transmissible Endosomal Intoxication (TEI)

The amyloidogenic hypothesis is at present the most important theory for the development of AD and places Aβ aggregates at the root of the pathology. Although a substantial body of evidence demonstrates that the Aß accumulation can lead to endolysosomal dysfunction, synapse loss, and cell death, the sequence of events is still under investigation. Especially since there are hallmarks of disease such as endolysosomal and autophagic dysfunctions that present much earlier and sometimes even decades before these Aβ aggregates. Not only do many genetic risk factors in AD directly tie to endolysosomal changes but also many environmental risk factors are also directly related to endolysosomal dysfunction. At a cellular level, this dysfunction can contribute to increased amyloidogenic processing of APP and to the generation of Aβ fibrils and oligomers by neurons and astrocytes. This dysfunction in microglia and likely neurons would lead to the accumulation of in situ generated or endocytosed Aβ and other APP cleavage products (especially C99). Cells would alleviate their congested endolysosomes by secreting them.

**Figure 3:**
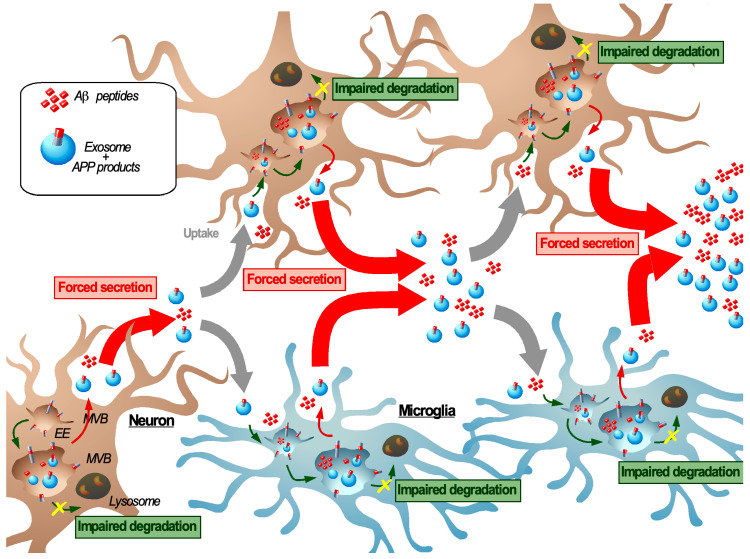
Transmissible endosomal intoxication. In neurodegenerative diseases such as Alzheimer′s disease, the imbalance between endosomal/autophagic degradation and secretion in neurons, induced or enhanced by C99 and Aβ_42_ accumulation promotes their release either or not in association with exosomes. As a mechanism of defense, neighboring cells (including neurons, here indicated in brown and microglia indicated in blue) can engulf these amyloids and exosomes by endocytosis, which induces a new cycle of endosomal congestion and re-secretion of amyloids and can lead to the intercellular propagation of endosomal intoxication. Here, we propose a novel model to describe this self-sustaining cycle; transmissible endosomal intoxication (TEI) as a way by which amyloids are propagated over the course of disease progression.

A first clearance pathway to eliminate amyloids from the intercellular space is by their uptake by neighboring cells especially scavenging microglia. However, a massive of uptake of amyloids would also lead to their subsequent endosomal accumulation and possibly to a re-secretion of the internalized material. This impaired balance between degradation and secretion in each individual cell may initiate a dynamic self-perpetuating process in which repeated cycles of internalization, endosomal congestion, and re-secretion of exosomes and amyloids from cell to cell lead to the spreading of AD-related cellular dysfunction throughout the brain (Figure 3). In analogy with the symptoms of food poisoning, we have called this process “transmissible endosomal intoxication” or TEI (Figure 3). This concept involves both organellar defects at an intracellular scale and an intercellular transmission at a cerebral scale. We pose that this TEI involves neurons (potentially different subtypes of neurons) as well as microglia cells, which all participate in the propagation of endosomal dysfunctions and amyloids from cell to cell. Importantly, this TEI establishes a functional relationship between two of the earliest features of AD: Endolysosomal dysfunction and APP cleavage product accumulation. However, the micro and/or macromolecular factors that underlie that onset sustenance of TEI are largely unknown and must be assessed, both for SAD and FAD.

The development of TEI suggests the involvement of the endolysosomal pathway in a transcellular proteostasis network (TPN). The TPN is in charge of maintaining proteostasis within tissues and organs under physiological conditions [185,186]. This process is based on the intercellular exchange of molecular chaperons as well as proteotoxic materials to support more vulnerable cells such as post-mitotic neurons and reveals a non-autonomous regulation of proteostasis. In this context, extracellular vesicles can serve as a vector to mediate the intercellular exchange [146]. Consequently, any flaws in this TPN would have the potential to contribute to TEI. This idea is in accordance with alterations of proteostasis with aging, which is the major risk factor for the most common sporadic forms of AD. Hence, a better understanding of intrinsic and extrinsic factors that regulate/affect the TPN might tell us how to counteract TEI.

This concept raises many new questions. In the context of amyloid-related disease an important point would be to elucidate the role of amyloids in this process. In what way do they contribute to the onset or sustenance of TEI? In other words, is TEI caused by the accumulation of amyloids or is it the result of an early defect of the TPN, which is then exacerbated by amyloids? These questions are particularly interesting in the light of the arguable contribution of Aβ in AD development and the failure of clinical trials using antibodies targeting Aβ. The role of exosomes in TEI also has to be elucidated, especially to determine their involvement in the dissemination of amyloids and to assess if they can directly contribute to TEI. The potential role of exosomes in TEI can raise other questions especially about the relative contribution of Aβ and C99 to TEI. As they possess different properties one can question if they act synergistically or if they each contribute at different stages in the progression of TEI? Along this line, a growing body of evidence suggests that APP cleavage products are transported by exosomes from neuron to neuron and from neuron to microglia and that exosomes can contribute to the AD pathology. Moreover, as the endosomal system is closely linked to macroautophagy, we can pose questions about the respective roles of classical lysosomal degradation and macroautophagy in TEI. Does their involvement depend on the cytosolic or endosomal location of amyloids and is the endolysosomal escape of amyloids through membrane damage required for TEI? This also provides a new angle to look at the contribution of the different cell types in the TEI. Which cell types are most sensitive to TEI and to what extent do neurons and microglia contribute to TEI after the uptake of amyloids and/or exosomes? Moreover, TEI could also contribute to other pathological phenotypes associated with AD such as neuroinflammation and a neuronal synaptic dysfunction.

To finish, the characterization of TEI requires determining precisely the progression of TEI and intrinsic and extrinsic factors that could affect it. TEI could be viewed as a dysfunction of the TPN and would reconcile the dual role of exosomes in the clearance (as part of the TPN) and the propagation of amyloids (as contributors of the TEI). At a cell biology level, it will be important to assess how the different regulators that are known to affect the balance between degradation and secretion (e.g., MVB sorting, the formation of MCS, and starvation) modulate TEI. Additionally it will be critical to study how it is affected by aging and by specific changes associated with AD susceptibility factors such as the *APOE4* isotype and inflammation. Future studies are required to further elucidate the mechanisms behind TEI in vivo disease models in order to define the factors that initiate its onset and cause its progression. This will potentially provide with new biomarkers and therapeutic leads that target this pathology from a different angle (Box 2). From a broader perspective these findings would then also have putative implications for other amyloid-associated pathologies that are associated with lysosomal defects.

Box 2 **Exosomes: Novel potential for biomarker utility and therapeutic applications in AD.**

Currently, AD specialists agree that one of the main reasons of AD clinical trial failure is the belated intervention, which is caused by the lack of reliable early biomarkers to diagnose AD at an early stage. Failure of clinical trials based on antibodies against Aβ could also suggest that the targeting of Aβ only could not be sufficient. An increasing knowledge about the biogenesis of exosomes during pathophysiological conditions has revealed that exosomes bear a unique signature that reflects the endolysosomal state of the donor cell and thus qualifies them as highly relevant candidates for the early diagnosis of AD. Additionally, their putative role in the maintenance of the TPN and their contribution to TEI also renders them as an interesting target for novel therapeutic applications against AD.

**Biomarkers**

The current diagnosis of AD is based on neuropsychological test and brain imaging. In addition, three AD biomarkers can be assessed upon cerebrospinal fluid (CSF) collection: Aβ_42_ and total and hyperphosphorylated Tau protein levels. However, these markers only appear relatively late in the course of disease and also increased with normal aging as well as for other neurodegenerative diseases, and thus further supports the urge to find more specific biomarkers that preferentially also require a less invasive method of collection. Exosomes contain a specific set of proteins and lipids reflective of the pathophysiological state of their cells of origin and hence provide potentially powerful biomarkers for an early diagnosis. As they are present in different body fluids (e.g., CSF, blood, and urine) they also form a non-invasive source of biomarkers in particular when they originate from poorly accessible organs or systems such as the central nervous system. Exosomes secreted by AD-neurons display a unique signature in terms of proteins (such as C99, Aβ, Tau, amyloid secretase, and Aβ-degrading enzymes) and lipids (including gangliosides and BMP). Interestingly, the presence of C99 in AD-neuron derived exosomes opens up the possibility to use this as an early biomarker. Indeed, mouse models of AD demonstrate that C99-containing exosomes appear at early stage of disease and similar observations have been made in AD patient brain tissues and CSF [32,56,187]. Similarly, lysosomal proteins levels (such as cathepsin D and LAMP-1) as well as autophagic proteins (e.g., p62) are altered in neuron-derived exosomes from AD models or patients, which is reflective of the lysosomal dysfunction that is associated with AD [64,137,188]. In this context, the establishment of such exosomal biomarkers would allow for an early diagnosis and the inclusion of early AD-patients in new clinical trials, targeting the endolysosomal and autophagic pathway for example. Similarly, it has been demonstrated that Aβ bound to exosomes proves a better biomarker than free Aβ allowing to distinguish AD from other neurodegenerative diseases [149]. Recent studies now show that neuron- and astrocyte-derived exosomes can be found in the circulation (plasma or serum) of AD patients, which means that the invasive approach for CSF collection can be omitted. Plasma neuron-derived exosomes also contain the AD biomarkers Aβ_42_ (elevated) and Tau (elevated), whose levels were found to be altered from as much as 10 years before the clinical onset allowing to distinguish between MCI and AD patients [189,190,191]. Similarly, Kapogiannis et al. have generated a large panel of plasma samples from healthy and cognitive impaired patients to develop a test for the prediction for AD, based on exosome protein profile [192]. Additionally, the exosomes content was also found to provide a way to follow the progression of the disease [190]. Astrocyte-derived exosomes from AD-patient derived samples also display a specific content and were found to have elevated BACE1 levels and pro-inflammatory cytokines (such as IL-6 and IL-1β) [193]. Moreover, several studies have demonstrated that also exosomal miRNAs can serve as a marker for AD [194,195]. Finally, other types of extracellular vesicles also qualify as a potential means for AD diagnosis, as microvesicles from AD patient CSF were also found to bear elevated Tau and APP levels [196]. Further characterization of exosomes from AD patient and healthy subject derived samples, based on stndardized isolation and analysis protocols, is required to establish how exosomes may aid to accurately differentiate between healthy and diseased patients.

**Therapeutic applications**

In addition to their clinical potential as biomarkers, exosomes can also serve as a novel therapeutic vector to interfere with AD, for which there are currently no therapeutics to prevent or reverse its progression. Indeed, their ability to cross the BBB and their inert nature makes them highly suitable for therapeutic applications. Several studies based on AD-mouse models have demonstrated the protective effect of the administration of exogeneous neuron-derived exosomes. Here, these vesicles were found to capture Aβ and to promote its degradation by microglia and thus to reduce the pathological phenotype associated with Aβ [54,151,197,198]. In this context, exosomes derived from mesenchymal stem cells (MSCs) were also proposed to have therapeutic potential and their beneficial effects have been demonstrated in several studies. Notably, adipose-derived MSCs have the capacity to secrete vesicles that contain the Aβ-degrading enzyme neprilysin, and thus may form another candidate for therapy [154]. Moreover, exosomes have been proposed as a vector for drug delivery and several studies have harnessed exosomes for targeted delivery to neurons [199] to equip them with a RNA packaging device, cytosolic delivery helper [200], or optogenetically driven loading of transcription factor and enzymes [201]. These engineered exosomes are then able to specifically target neurons where they can decrease the expression of BACE1 [199] yet their relevance for AD treatment remains to be demonstrated. With the aim to boost lysosomal biogenesis, exosomes have also been primed with curcumin, an activator of TFEB [202]. However, exosomes based therapy still proves rather inefficient as the vast majority is cleared before reaching their target. Hence, the therapeutic use of exosomes requires a more extensive investigation with regard to their safety, their stability, their biodistribution and how this is affected by the mode of administration. To do so, the field needs to develop novel approaches that allow to track exosomes at a single vesicle scale [203].

An alternative strategy would be to target the biogenesis of endogenous exosomes in order to modulate their composition or secretion in order to diminish exosome-associated amyloid aggregation, propagation, and associated cytotoxicity. As mentioned in chapter 5 describing the role of exosome in AD pathogenesis, “alternative” exosomes enriched in atypical lipids such as ceramide and specific gangliosides as well as C99 may contribute to the AD pathology a.o. by their ability to promote Aβ aggregation. Importantly, a study that demonstrates how endolysosomal pathway aberrations induce the release of these C99 containing vesicles also shows that the release of these vesicles was inhibited by GW4869 and myriocin [73,159]. GW4869 is a neutral sphingomyelinase inhibitor that inhibits the formation of vesicles from ceramide rich lipid rafts and myriocin inhibits the enzyme that catalyzes the first step in the sphingolipid synthesis. Both compounds would act in the formation or secretion of ILVs as they reduce the release of Alix, flotillin-2, Tsg101, GM1 ganglioside, C99, and poly-ubiquitin positive vesicles. GW4869 treatment cannot only prevent Aβ aggregation, in vitro, but also in vivo it was found to reduce the levels of Aβ_42_ as well as the Aβ plaque load and to improve cognition [73,159]. Moreover, also the sphingomyelinase inhibitor desipramine which, similar to GW4869 and myriocin, reduces the release of vesicles, was found to improve clinical symptoms in AD patients including depression-like behavior and working memory [204]. Thus, targeting the exosome biogenesis by sphingomyelinase inhibition may provide an attractive strategy to reduce the amyloid burden, yet further studies would be required to assess its safety for long term use as this would not only affect the release of exosomes but would also have a broader effect on various other cellular functions.
